# Reliability and Construct Validity of the Communication Function Classification System (CFCS) in an Italian Population of Children with Cerebral Palsy

**DOI:** 10.3390/children13010012

**Published:** 2025-12-20

**Authors:** Azzurra Speroni, Gessica Della Bella, Antonella Cerchiari, Ester Mignolli, Marco Tofani

**Affiliations:** 1 Management and Diagnostic Innovations & Clinical Pathways Research Area, Neurorehabilitation and Adapted Physical Activity Day Hospital, Bambino Gesù Children’s Hospital, 00165 Rome, Italy; azzurra.speroni@opbg.net (A.S.); gessica.dellabella@opbg.net (G.D.B.); antonella.cerchiari@opbg.net (A.C.); ester.mignolli@opbg.net (E.M.); 2 Department of Life Sciences, Health and Healthcare Professions, Università degli Studi “Link Campus University”, 00165 Rome, Italy

**Keywords:** cerebral palsy, communication, CFCS, GMFCS, validation, reliability, construct validity, rehabilitation

## Abstract

**Background/objectives:** Communication difficulties are highly prevalent among children with cerebral palsy (CP) and have a significant impact on participation, psychosocial development, and quality of life. The Communication Function Classification System (CFCS) was developed to provide a standardized framework for describing functional communication performance across five ordinal levels. While the CFCS has been validated internationally, evidence on its psychometric properties in Italian pediatric populations remains limited. The objective of this study was to examine the inter-rater and intra-rater reliability of the Italian version of the CFCS and to explore construct validity through a single association with the Gross Motor Function Classification System Expanded and Revised (GMFCS E&R). **Methods**: A cross-sectional study was conducted with 66 children with CP (mean age 8.8 years, SD = 4.9) recruited from the Bambino Gesù Children’s Hospital in Rome. Two trained raters independently classified each child using the CFCS and GMFCS E&R, with CFCS reassessments performed after 14–20 days to evaluate intra-rater stability. Agreement was assessed using linear weighted Cohen’s Kappa (κᵂ) coefficients, and construct validity was analyzed using Spearman rho correlation (r) between CFCS and GMFCS E&R levels. **Results**: The CFCS demonstrated almost very good agreement for both inter-rater and intra-rater reliability, with κᵂ values exceeding 0.90. Construct validity was supported by a strong and statistically significant correlation with GMFCS E&R (r = 0.82, *p* < 0.01), indicating that greater motor impairment was associated with more severe communication limitations. **Conclusions**: The Italian version of the CFCS is a highly reliable classification system and shows evidence of construct validity based on a single convergent association in children with CP. These findings support its use for descriptive and classificatory purposes in clinical and research contexts, while further studies are needed to examine additional psychometric properties.

## 1. Introduction

Communication is a fundamental human function, essential for social interaction, learning, and participation in daily life. It encompasses not only the transmission of information but also the dynamic exchange of meanings, emotions, and intentions between individuals. In rehabilitation sciences, communication is considered a multidimensional construct that involves both expressive and receptive aspects, relying on verbal and non-verbal modalities as well as contextual and relational factors. When communication is impaired, the consequences extend beyond linguistic deficits, profoundly affecting participation, psychosocial development, and quality of life [1].

A distinction must be made between verbal communication and communication function. Verbal communication primarily refers to speech production, intelligibility, and language structures, whereas communication function encompasses the overall effectiveness with which an individual can participate in communicative exchanges in everyday contexts. This includes gestures, facial expressions, augmentative and alternative communication (AAC) [2], and the individual’s ability to alternate effectively between the role of sender and receiver. From a rehabilitation standpoint, communication function is therefore a broader construct, aligned with the World Health Organization’s International Classification of Functioning, Disability and Health (ICF) [3], which emphasizes activity and participation rather than impairment alone [4].

In children with cerebral palsy (CP), communication difficulties are highly prevalent: epidemiological studies report that approximately half of children with CP have speech/language or broader communication impairments, while nearly one-quarter are classified as non-speaking [5], particularly among children with greater motor and cognitive involvement [6]. The result is that many children with CP risk assuming a passive role in communication, where conversational partners dominate interactions and opportunities for active participation are restricted. These challenges are particularly significant given that CP is the most common cause of physical disability in childhood, with prevalence estimates of 1.6 per 1000 live births in high-income countries, while in low- and middle-income countries the prevalence is higher [7]. Given the heterogeneity of CP, a classification of communication function is necessary to describe differences among children, to guide clinical decision-making, and to enable the comparison of data across studies. The ICF framework provides a conceptual basis for such classification, as it highlights not only body functions and structures but also activity, participation, and contextual factors. A standardized measure of communication function allows clinicians and researchers to stratify samples, design targeted interventions, and monitor outcomes with comparable metrics.

The Communication Function Classification System (CFCS), developed by Hidecker and colleagues in 2011 [8], was introduced to meet this need. The CFCS is a five-level ordinal scale that classifies everyday communication performance in individuals with CP, taking into account both sending and receiving roles, conversational pace, and partner familiarity. Unlike tools limited to speech or language assessment, the CFCS evaluates the effectiveness of communication in natural contexts, regardless of the modality employed. It therefore captures the range of communicative strategies—including AAC—that children may use, offering a clinically relevant perspective on participation.

Since its development, the CFCS has undergone several validation studies internationally [9] and have confirmed strong correlations between CFCS levels and other functional classification systems, such as the Gross Motor Function Classification System (GMFCS) and the Manual Ability Classification System (MACS) [9,10]. The standardization of communication classification is crucial for both clinical and research purposes. Clinically, it provides a common language among healthcare professionals, caregivers, and educators, facilitating goal-setting and intervention planning. In research, it enables comparability across studies and supports stratification of samples in clinical trials, longitudinal follow-ups, and outcome evaluations. Without standardized functional classifications, data remain fragmented, limiting the generalizability of findings and the development of evidence-based guidelines. Despite growing international evidence, Italian studies using the CFCS remain limited, particularly with regard to formal psychometric evaluation and the examination of its relationship with other functional domains. To date, few studies in Italian samples have specifically investigated the association between communication function, as classified by the CFCS, and motor function or other functional parameters in children with CP [11,12]. Establishing the reliability of the CFCS in Italy is essential to ensure that clinicians and researchers can apply it with confidence in both clinical practice and research studies. Moreover, exploring the relationship between communication and motor abilities may further clarify the interplay between functional domains in CP, supporting integrated rehabilitation planning.

The present study aimed to measure the inter-rater and intra-rater reliability of the CFCS in a sample of Italian children with CP and to examine its construct validity by exploring the correlation between communication function and gross motor abilities as classified by the GMFCS E&R. We hypotheses that (a) the CFCS is a reliable tool for classifying communication function in Italian children with CP, showing high inter-rater and intra-rater agreement; and (b) communication and motor functions are positively correlated, with higher levels of motor impairment associated with greater communication difficulties.

## 2. Materials and Methods

### 2.1. Study Design

This was a cross-sectional observational study designed to evaluate the psychometric properties of the CFCS in a pediatric Italian population with CP. The research groups have solid experience in validation of outcome measures and developmental disabilities [13,14,15]. The study was approved by the Ethics Committee of Bambino Gesù Children’s Hospital with protocol number 3329_OPBG_2024.

### 2.2. Participants

An a priori sample size estimation for the construct validity analysis was performed assuming a minimum relevant monotonic association of r = 0.40 between CFCS and GMFCS E&R, corresponding to a moderate and clinically plausible correlation. With a two-tailed significance level of α = 0.05 and 80% power, the required sample size was estimated using Fisher’s z transformation, resulting in a minimum of 47 participants. Children were recruited through a convenience sampling strategy from the Bambino Gesù Children’s Hospital IRCCS (Scientific Institute for Research, Hospitalization and Healthcare). Recruitment was carried out over a 6-month period. Eligibility was restricted to children with a confirmed diagnosis of CP according to the Surveillance of Cerebral Palsy in Europe (SCPE) criteria [16], aged between 0 and 18 years, and with sufficient familiarity with the Italian language to ensure that caregivers and raters could provide reliable responses. Children with cerebral visual impairment or significant hearing impairment were excluded, as these conditions could have independently affected communication performance and confounded the interpretation of communication-related outcomes. In contrast, children with mild visual impairments and children with cognitive impairment were not excluded, provided that they were able to participate in the assessment and that caregivers could meaningfully report on communication abilities. Children were also excluded if they presented with neurological or genetic conditions other than CP, if they were hospitalized in intensive or sub-intensive care units at the time of assessment, or if their clinical status was unstable due to severe medical complications, unconsciousness, or sedation, which would have prevented the evaluation of communication abilities.

### 2.3. Instruments

#### 2.3.1. Communication Function Classification System (CFCS)

The CFCS is a five-level ordinal scale originally developed by Hidecker and colleagues [8] to provide a standardized framework for describing everyday communication performance in individuals with CP. For this study, the Italian translation available on the official CFCS website was adopted, ensuring linguistic and cultural consistency with the target population. The CFCS is designed to classify functional communication based on several key dimensions, namely the child’s effectiveness as both sender and receiver of messages, the fluency and pace of conversational exchanges, and the ability to engage with familiar and unfamiliar communication partners. Importantly, the classification does not focus solely on verbal language, but rather considers the full spectrum of communicative modalities, including spoken speech, gestures, facial expressions, and AAC strategies. The five levels of the CFCS represent a continuum of communication performance in daily life. At the highest level (Level I), children are able to communicate effectively as both senders and receivers with familiar and unfamiliar partners, showing fluid exchanges with minimal breakdowns. Level II also reflects effective communication with all partners, but interactions are characterized by a slower rhythm, with increased time needed for message formulation, comprehension, or repair. At Level III, effectiveness is maintained only with familiar partners, whereas interactions with strangers or less familiar individuals are less reliable or inconsistent. Children classified at Level IV show irregular effectiveness even when communicating with familiar partners, alternating between successful and unsuccessful exchanges. Finally, Level V identifies children whose communication is seldom effective, even with partners who know them well, indicating substantial limitations in both sending and receiving roles across daily contexts. The Italian version of the CFCS used in this study corresponds to the official translation made available on the CFCS website (please see www.cfcs.us), which hosts authorized versions of the instrument in multiple languages for international use. These translations are disseminated by the CFCS developers and are intended to support standardized application of the classification system across different linguistic and cultural contexts.

#### 2.3.2. Gross Motor Function Classification System—Expanded & Revised (GMFCS E&R)

The GMFCS E&R [17] is one of the most widely adopted functional classification tools in the field of CP. Developed to provide a standardized description of gross motor abilities, it categorizes children into five ordinal levels according to their typical performance in everyday environments, rather than their best capacity under ideal circumstances. The GMFCS E&R takes into account the child’s ability to sit, stand, transfer, and move across home, school, and community settings, with particular emphasis on mobility and the use of assistive devices such as walkers, crutches, or wheelchairs [18]. The five levels of the GMFCS E&R describe a continuum of motor performance that ranges from children who are able to walk without limitations (Level I) to those who are transported in a manual wheelchair (Level V). Intermediate levels capture the use of walking aids, limitations in outdoor or long-distance mobility, and the reliance on powered mobility devices when independent ambulation is no longer feasible. The classification is designed to reflect functional mobility across different ages, and the expanded version incorporates developmental expectations up to 18 years of age, thus allowing for longitudinal consistency in clinical and research contexts.

### 2.4. Procedures

Prior to the initiation of data collection, two rehabilitation professionals—an occupational therapist and a speech and language therapist—completed a structured training program on the use of both the CFCS and the GMFCS E&R. The training comprised a review of the official manuals, examination of case examples, and a series of consensus exercises designed to promote a uniform interpretation of the classification criteria and to reduce potential sources of rater variability. The calibration process was organized into two sessions of approximately 90 min each and involved the joint review of eight clinical cases selected to represent the full range of communication profiles encompassed by the CFCS. The sessions were supervised by a senior clinician with longstanding experience in the application of functional classification systems in CP, who oversaw discussions and ensured agreement on the operationalization of each descriptor. This preparatory work was essential to harmonize the evaluators’ approach and likely contributed to the high reliability values observed.

Assessments were carried out in clinical environments that were already familiar to the children, such as hospital outpatient or inpatient settings, thereby reducing the risk of situational anxiety or atypical behavior. Whenever appropriate, parents or primary caregivers were present during the evaluations, and their input was actively sought. In particular, for the CFCS, caregivers were asked to provide examples of the child’s everyday communication strategies and contexts, supplementing the direct observations of the raters. To limit the influence of short-term variability factors such as fatigue, mood, or transient health conditions, assessments were conducted during routine clinical appointments and, as far as possible, at similar times of day for each child. Evaluations were scheduled to avoid periods immediately following demanding therapeutic sessions or medical procedures. This approach aimed to ensure that CFCS ratings reflected the child’s habitual communication performance in daily life rather than temporary fluctuations or isolated best performances observed in a clinical setting.

Each participant was independently classified by the two raters [19] using both the CFCS and the GMFCS E&R. For inter-rater reliability, the two clinicians performed their classifications independently during the same assessment phase, without discussion and without access to each other’s ratings; CFCS scores were recorded separately for each rater. For the CFCS, the evaluation considered the child’s effectiveness in assuming both the roles of sender and receiver, the pace of conversational turn-taking, and the capacity to interact with both familiar and unfamiliar partners, regardless of the communicative modality employed (speech, gestures, facial expressions, or augmentative and alternative communication devices). For the GMFCS E&R, classification was based on the child’s usual mobility performance across home, school, and community contexts, with emphasis on sitting, standing, transfers, and the use of assistive devices. To examine intra-rater reliability, the CFCS was re-administered by the same raters after an interval of 14–20 days. This timeframe was chosen deliberately: it was sufficiently short to minimize developmental changes or intervention effects that could alter communication performance, but sufficiently long to reduce the likelihood that raters would recall their previous classification. At the time of reassessment, raters did not have access to their previous CFCS ratings, and no records of prior classifications were available during the second assessment. Data were systematically collected through a structured electronic form created in Google Forms. The form was organized into sections, beginning with demographic variables such as age, sex, and CP subtype, and followed by the classification outcomes for both CFCS and GMFCS E&R. This digital format facilitated the standardization of data entry, minimized transcription errors, and enabled efficient data export for subsequent statistical analysis.

### 2.5. Statistical Analysis

Data analysis was performed using SPSS Statistics version 27 (IBM Corp., Armonk, NY, USA). Descriptive statistics, including means and standard deviations for continuous variables and frequencies and percentages for categorical variables, were calculated to provide a comprehensive overview of participant demographics and the distribution of classification outcomes across both the CFCS and the GMFCS E&R.

The assessment of reliability focused on both intra- and inter-rater agreement for the CFCS. Given the ordinal and categorical structure of the classification system, reliability was evaluated using linear weighted Cohen’s Kappa, which measures the degree of concordance between raters beyond chance [20]. Linear weighting was selected as the primary reliability index because it accounts for the ordered structure of the scale and provides a conservative estimate of agreement by assigning proportionally greater penalties to disagreements between more distant categories. Percentage agreement was also calculated as a complementary measure to facilitate interpretation, particularly in the presence of unbalanced category distributions. Values were interpreted according to conventional benchmarks, with coefficients below 0.40 considered indicative of poor agreement, values between 0.41 and 0.60 reflecting moderate agreement, values between 0.61 and 0.80 indicating substantial agreement, and values greater than 0.80 denoting almost perfect agreement [21,22]. Construct validity was investigated by examining the association between CFCS and GMFCS E&R. Spearman rho correlation coefficient (r) was calculated to assess the linear relationship between the two ordinal variables, as both scales follow a monotonic progression of functional ability [23]. Correlation strength was interpreted as weak when r ranged from 0.0 to 0.3, moderate when values ranged from 0.3 to 0.7, and strong when exceeding 0.7 [24]. Statistical significance was set at *p* < 0.05, two-tailed. In addition, scatterplots and box-plots were generated to provide a visual representation of the relationship between motor and communication function, facilitating the interpretation of correlations across the spectrum of functional levels.

## 3. Results

We recruited a total of 66 children with CP, most of whom presented with the bilateral spastic form (54.5%), followed by dyskinetic (31.8%). The distribution across GMFCS indicated a predominance of children with more severe motor impairments, as Levels IV and V each accounted for 33.3% of the sample. Similarly, according to the CFCS, higher severity levels (Levels IV–V) were common, representing more than half of the participants. The main demographic and clinical characteristics of the study population are summarized in Table 1.

The distribution of CFCS levels across raters and time points demonstrated consistent classification patterns, with minimal variation between assessments. Excellent agreement was observed both for intra-rater reliability (κᵂ = 0.95, 95% CI: 0.91–0.99) and inter-rater reliability (κᵂ = 0.91, 95% CI: 0.83–0.98), with a percentage agreement of 93.9% and 90.9%, respectively. Detailed distributions and agreement coefficients are reported in Table 2.

To provide a more detailed representation of rating concordance, the contingency tables underlying the reliability analyses are reported in Table 3 and Table 4. Table 3 presents the cross-tabulation of CFCS levels assigned at Time 1 and Time 2 by the same rater for the intra-rater reliability assessment, while Table 4 shows the cross-tabulation of CFCS levels independently assigned by Rater A and Rater B for the inter-rater reliability assessment. These tables display the frequency distribution of CFCS classifications across assessments and raters, allowing visualization of exact agreement along the diagonal and the distribution of any discrepancies across adjacent and non-adjacent levels.

**Figure 1:**
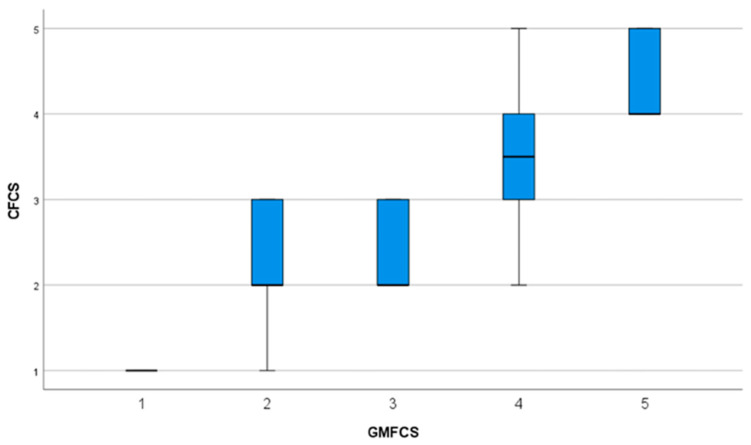
Box-plot illustration for GMFCS and CFCS.

Construct validity analysis revealed a Spearman rho coefficient of 0.82, 95% CI 0.72–0.89 (*p* < 0.01). Figure 1 illustrates the relationship between GMFCS E&R and CFCS levels, showing a clear monotonic trend whereby higher levels of gross motor impairment are associated with more severe communication limitations. Lower GMFCS levels are predominantly associated with lower CFCS levels, whereas children classified at GMFCS Levels IV and V are more frequently classified within the higher CFCS categories. Notably, greater variability in CFCS levels is observed at intermediate GMFCS levels, indicating that children with similar motor severity may present heterogeneous communication functioning. This pattern is further detailed in the contingency table (Table 5), which reports the distribution of CFCS levels across GMFCS categories and highlights the overlap between communication and motor classifications, particularly in the mid-range of functional severity.

## 4. Discussion

The present study aimed to evaluate the measurement properties of the Italian version of the CFCS in a sample of children with CP. The main findings demonstrate that the CFCS has excellent intra-rater and inter-rater reliability, with κᵂ coefficients of 0.95 and 0.91, respectively, and strong construct validity, as evidenced by the significant correlation with the GMFCS E&R (r = 0.82, *p* < 0.01). These results indicate that the CFCS can be considered a reliable and valid tool for assessing communication function in Italian children with CP, suitable for both clinical practice and research purposes.

Our findings are consistent with the original validation study by [8] and colleagues, which reported κᵂ values ranging from 0.66 to 0.92 among raters from different professional backgrounds. The high level of agreement observed in our sample falls within the upper range of those estimates, supporting the cross-cultural robustness of the CFCS classification criteria. Similar results have been reported in other linguistic adaptations. For instance, Mutlu et al. [25] found inter-rater intraclass correlation coefficients (ICCs) between 0.95 and 0.96 in a Turkish cohort of 102 children, while Soleymani and colleagues [26] observed intra-rater ICCs of 0.94–0.98 and inter-rater ICCs of 0.74–0.88 in a Persian-speaking population. Likewise, Choi [27] confirmed high consistency between therapists and caregivers in a Korean sample, with weighted Kappa values above 0.80. The comparable reliability levels across countries suggest that the CFCS operational definitions are sufficiently clear and that the scale captures universal features of communication functioning in children with CP, regardless of linguistic or cultural background.

In our study, the high agreement between raters can be attributed to several methodological factors. Firstly, both raters were trained rehabilitation professionals—a speech and language therapist and an occupational therapist—who underwent a structured calibration process before data collection. This approach ensured conceptual alignment and minimized interpretive variability, an aspect that may explain the slightly higher reliability values compared with earlier studies that involved raters with more heterogeneous expertise. Secondly, the time interval adopted for intra-rater testing (14–20 days) was adequate to minimize recall bias while preventing potential changes in communication performance due to therapeutic interventions or developmental progression. This methodological rigor strengthens the evidence for the temporal stability of the Italian CFCS and supports its reproducibility for use in repeated assessments, longitudinal follow-ups, and outcome evaluations.

Regarding construct validity, the strong correlation between CFCS and GMFCS E&R (r = 0.82) supports the theoretical hypothesis that communication and gross motor functions are closely interrelated domains in children with CP. This relationship has been consistently demonstrated in previous studies, though with varying magnitudes of correlation (r ranging from 0.40 to 0.86) [25,26,28,29]. Our correlation value lies at the higher end of this spectrum, reinforcing the notion that more severe motor impairments are often accompanied by greater communication limitations. Several neurodevelopmental mechanisms may explain this association. Motor and communication functions rely on overlapping cortical and subcortical networks, particularly in regions involved in motor planning, sensorimotor integration, and orofacial control. Lesions affecting these networks can simultaneously compromise gross motor and speech-motor functions, resulting in both mobility and communication deficits. Furthermore, children with severe motor impairments often experience restricted opportunities for social interaction, reduced environmental exploration, and limited access to alternative communication modalities, all of which may negatively impact communicative competence and participation.

Nevertheless, while motor and communication functions are related, they remain distinct constructs reflecting different aspects of functioning. The GMFCS focuses on movement and mobility across contexts, while the CFCS measures communicative effectiveness, encompassing both expressive and receptive modalities and the ability to adapt across familiar and unfamiliar partners. The strong but non-overlapping correlation observed in this study confirms that each classification system provides complementary information, aligning with the World Health Organization’s International Classification of Functioning, Disability and Health (ICF) framework [30]. This multidimensional perspective is crucial for clinical practice, as it underscores the need to assess communication alongside motor, manual, and cognitive abilities to obtain a comprehensive profile of functioning. The integration of CFCS and GMFCS data can thus guide more holistic intervention planning, facilitate goal setting within multidisciplinary teams, and enhance communication between healthcare professionals, educators, and families.

Another relevant finding concerns the distribution of CFCS levels in our cohort, which was skewed toward the more severe categories (Levels IV–V). This reflects the recruitment setting—a tertiary pediatric rehabilitation hospital specializing in complex neurological conditions—and is consistent with epidemiological data indicating that children with bilateral spastic and dyskinetic CP, who constituted the majority of our sample, are more likely to experience significant communication challenges. At the same time, this severity distribution does not fully represent the broader Italian CP population, in which milder motor and communication profiles are proportionally more common and typically managed in community-based services. The predominance of children at the extreme ends of the CFCS scale may also have influenced the psychometric results: classifications at the margins are generally less ambiguous, which may partly explain the high intra- and inter-rater reliability observed. Likewise, the strong correlation between CFCS and GMFCS may have been amplified by reduced variability and clustering of severe functional profiles, as both measures capture the cumulative burden of motor and communication limitations within this clinical population. These considerations highlight the need for future multicenter studies with a more balanced distribution of severity levels to improve generalizability and refine estimates of construct validity.

Despite these strengths, several limitations should be acknowledged. First, the sample size, while comparable to other validation studies, was limited to a single rehabilitation center and may not fully represent the broader Italian CP population, including children with milder forms of impairment or those receiving community-based services. Second, CFCS classifications in the present study were based exclusively on clinician ratings. Although caregiver input was used to contextualize everyday communication performance, caregivers did not provide independent CFCS classifications, and parent–clinician agreement was not examined. Previous studies have emphasized the relevance of caregiver ratings for ecological validity, and this aspect should be addressed in future research. Third, although the time interval for intra-rater testing was carefully chosen, external factors such as fatigue, mood, or daily variability in communicative behavior could have influenced individual ratings. Fourth, construct validity was examined only through correlation with the GMFCS E&R, which primarily reflects gross motor functioning and therefore captures only one dimension of overall functioning in children with CP. While the observed association supports the expected relationship between motor and communication domains, reliance on a single external classification system may limit the comprehensiveness of construct validity assessment. Additional validation against other functional measures, such as the Manual Ability Classification System (MACS) [31,32], Eating and Drinking Abilities Classification System (EDACS) [33,34,35] and Visual Function Classification System (VFCS) [36] or cognitive assessments, would provide a more nuanced understanding of the CFCS’s convergent and discriminant properties. Future research should therefore expand on these findings by including larger and more diverse samples across multiple clinical settings, encompassing the full spectrum of CP severity and subtypes. Moreover, integrating parental ratings alongside professional classifications may provide valuable insights into ecological validity and real-world applicability.

## 5. Conclusions

The Italian version of the CFCS demonstrated excellent reliability and evidence of construct validity based on a single convergent association in a clinical sample of children with CP. The high intra- and inter-rater agreement values confirm the tool’s consistency across raters and time, while the strong correlation with the GMFCS E&R supports a convergent relationship between communication and motor functioning, underscoring their functional interdependence. The CFCS provides a standardized, user-friendly framework for describing communication performance across everyday contexts, supporting its implementation in both clinical rehabilitation and research environments. While further studies are needed to examine additional psychometric properties, its integration into multidisciplinary assessment protocols can facilitate shared decision-making, enhance goal-oriented care, and contribute to the development of evidence-based strategies aimed at improving participation and quality of life for children with CP and their families.

## Figures and Tables

**Table 1:** Sample Characteristics.

Category	Value
Age (years), mean (SD)	8.8 (4.9)
Sex	n (%)
Male	36 (54.5%)
Female	30 (45.5%)
SCPE	
Bilateral spastic	36 (54.5%)
Dyskinetic	21 (31.8%)
Ataxic	5 (7.6%)
Unilateral spastic	4 (6.1%)
CFCS	
Level I	2 (3.0%)
Level II	14 (21.2%)
Level III	17 (25.8%)
Level IV	14 (21.2%)
Level V	19 (28.8%)
GMFCS E&R n (%)	
Level I	1 (1.5%)
Level II	6 (9.1%)
Level III	15 (22.7%)
Level IV	22 (33.3%)
Level V	22 (33.3%)

Abbreviations: CFCS = Communication Function Classification System; GMFCS E&R = Gross Motor Function Classification System Expanded and Revised; SD = standard deviation.

**Table 2:** Inter- and intra-rater reliability of the CFCS (tot 66).

CFCS Level	T1/Rater A n (%)	T2 n (%)	Rater B n (%)
1	2 (3.0)	2 (3.0)	2 (3.0%)
2	14 (21.2)	15 (22.7)	14 (21.2%)
3	17 (25.8)	16 (24.2)	16 (24.2)
4	14 (21.2)	15 (22.7)	14 (21.2)
5	19 (28.8)	18 (27.3)	20 (30.3)
**Agreement κᵂ 95% CI**			
Intra-rater (T1 vs. T2)	0.95 (0.91–0.99 95% CI)		
Inter-rater (A vs. B)	0.91 (0.83–0.98 95% CI)		

T1–T2: Time administration 1 and 2; A and B: Rater A and B; CFCS: Communication Function Classification System.

**Table 3:** Intra-rater contingency table of CFCS classifications at Time 1 and Time 2.

CFCS							
	T2	I	II	III	IV	V	Total
T1							
**I**		2	0	0	0	0	**2**
**II**		0	14	0	0	0	**14**
**III**		0	1	15	1	0	**17**
**IV**		0	0	1	13	0	**14**
**V**		0	0	0	1	18	**19**
**Total**		**2**	**15**	**16**	**15**	**18**	**66**

**Table 4:** Inter-rater contingency table of CFCS classifications assigned by Rater A and B.

CFCS							
	A	I	II	III	IV	V	Total A
B							
**I**		2	0	0	0	0	**2**
**II**		0	14	0	0	0	**14**
**III**		0	0	14	2	1	**17**
**IV**		0	0	1	12	1	**14**
**V**		0	0	1	0	18	**19**
**Total B**		**2**	**14**	**16**	**14**	**20**	**66**

**Table 5:** Cross-tabulation of CFCS levels across GMFCS E&R levels.

	CFCS	I	II	III	IV	V	Total CFCS
GMFCS							
**I**		1	1	0	0	0	**2**
**II**		0	4	8	2	0	**14**
**III**		0	1	6	7	3	**17**
**IV**		0	0	1	6	7	**14**
**V**		0	0	0	7	12	**19**
**Total GMFCS**		**1**	**6**	**15**	**22**	**22**	**66**

## Data Availability

The data presented in this study are available on request from the corresponding author. The data are not publicly available due to privacy and ethical reasons.

## References

[1] Duinmeijer I., Peet S., Janssen L., Scheper A., Zwitserlood-Nijenhuis M., Bliekendaal W., Zoons M., Hakvoort B. (2025). Language, Communicative Participation, and Well-Being in Young Children with (Presumed) Developmental Language Disorder. Int. J. Lang. Commun. Disord..

[2] Tsai M.-J. (2022). Using the ICF Framework to Assess Communicative Competence in Dyadic Communication among Children and Adolescents Who Use Augmentative and Alternative Communication Devices in Taiwan. Behav. Sci..

[3] World Health Organization (2001). The ICF: An Overview.

[4] Threats T., Worrall L. (2004). Classifying Communication Disability Using the ICF. Adv. Speech Lang. Pathol..

[5] Mei C., Reilly S., Reddihough D., Mensah F., Pennington L., Morgan A. (2016). Language Outcomes of Children with Cerebral Palsy Aged 5 Years and 6 Years: A Population-based Study. Dev. Med. Child Neurol..

[6] Zhang J.Y., Oskoui M., Shevell M. (2015). A Population-Based Study of Communication Impairment in Cerebral Palsy. J. Child. Neurol..

[7] McIntyre S., Goldsmith S., Webb A., Ehlinger V., Hollung S.J., McConnell K., Arnaud C., Smithers-Sheedy H., Oskoui M., Khandaker G. (2022). Global Prevalence of Cerebral Palsy: A Systematic Analysis. Dev. Med. Child Neurol..

[8] Hidecker M.J.C., Paneth N., Rosenbaum P.L., Kent R.D., Lillie J., Eulenberg J.B., Chester K., Johnson B., Michalsen L., Evatt M. (2011). Developing and Validating the Communication Function Classification System for Individuals with Cerebral Palsy. Dev. Med. Child Neurol..

[9] Piscitelli D., Ferrarello F., Ugolini A., Verola S., Pellicciari L. (2021). Measurement Properties of the Gross Motor Function Classification System, Gross Motor Function Classification System-Expanded & Revised, Manual Ability Classification System, and Communication Function Classification System in Cerebral Palsy: A Systematic Review with Meta-analysis. Dev. Med. Child Neurol..

[10] Palisano R.J., Avery L., Gorter J.W., Galuppi B., McCoy S.W. (2018). Stability of the Gross Motor Function Classification System, Manual Ability Classification System, and Communication Function Classification System. Dev. Med. Child Neurol..

[11] Pagliano E., Casalino T., Mazzanti S., Bianchi E., Fazzi E., Picciolini O., Frigerio A., Rossi A., Gallino F., Villani A. (2021). Being Adults with Cerebral Palsy: Results of a Multicenter Italian Study on Quality of Life and Participation. Neurol. Sci..

[12] Compagnone E., Maniglio J., Camposeo S., Vespino T., Losito L., De Rinaldis M., Gennaro L., Trabacca A. (2014). Functional Classifications for Cerebral Palsy: Correlations between the Gross Motor Function Classification System (GMFCS), the Manual Ability Classification System (MACS) and the Communication Function Classification System (CFCS). Res. Dev. Disabil..

[13] Cerchiari A., Tofani M., Giordani C., Franceschetti S., Capuano E., Pizza F., Della Bella G., Raponi M., Biondo G. (2023). Development and Pilot Study of a Pediatric Screening for Feeding and Swallowing Disorders in Infants and Children: The Pediatric Screening–Priority Evaluation Dysphagia (PS–PED). Children.

[14] Cerchiari A., Pizza F., Biondo G., Giordani C., De Paolis M., Della Bella G., Raponi M., Tofani M. (2025). Evaluating the Global Intensive Feeding Therapy (GIFT) for Children with CHARGE Syndrome: A Quasi-Experimental Study. Children.

[15] Pizza F., Tofani M., Biondo G., Giordani C., Murgioni C., Raponi M., Della Bella G., Cerchiari A. (2025). Translation, Cross-Cultural Adaptation and Validation of the Karaduman Chewing Performance Scale for the Italian Paediatric Population. J. Evaluation Clin. Pr..

[16] Cans C. (2000). Surveillance of Cerebral Palsy in Europe: A Collaboration of Cerebral Palsy Surveys and Registers. Dev. Med. Child Neurol..

[17] McDowell B. (2008). The Gross Motor Function Classification System—Expanded and Revised. Dev. Med. Child Neurol..

[18] Morris C., Orth S.R., Bartlett D. (2004). Gross Motor Function Classification System: Impact and Utility. Dev. Med. Child Neurol..

[19] Monticone M., Galeoto G., Berardi A., Tofani M. (2021). Psychometric Properties of Assessment Tools. Measuring Spinal Cord Injury.

[20] Sim J., Wright C.C. (2005). The Kappa Statistic in Reliability Studies: Use, Interpretation, and Sample Size Requirements. Phys. Ther..

[21] Li M., Gao Q., Yu T. (2023). Kappa Statistic Considerations in Evaluating Inter-Rater Reliability between Two Raters: Which, When and Context Matters. BMC Cancer.

[22] O’Leary S., Lund M., Ytre-Hauge T.J., Holm S.R., Naess K., Dalland L.N., McPhail S.M. (2014). Pitfalls in the Use of Kappa When Interpreting Agreement between Multiple Raters in Reliability Studies. Physiotherapy.

[23] Akoglu H. (2018). User’s Guide to Correlation Coefficients. Turk. J. Emerg. Med..

[24] Sedgwick P. (2014). Spearman’s Rank Correlation Coefficient. BMJ.

[25] Mutlu A., Kara Ö.K., Livanelioğlu A., Karahan S., Alkan H., Yardımcı B.N., Hidecker M.J.C. (2018). Agreement between Parents and Clinicians on the Communication Function Levels and Relationship of Classification Systems of Children with Cerebral Palsy. Disabil. Health J..

[26] Soleymani Z., Joveini G., Baghestani A.R. (2015). The Communication Function Classification System: Cultural Adaptation, Validity, and Reliability of the Farsi Version for Patients With Cerebral Palsy. Pediatr. Neurol..

[27] Choi J.Y., Hwang E.H., Rha D., Park E.S. (2018). Reliability and Validity of the Korean-language Version of the Communication Function Classification System in Children with Cerebral Palsy. Child. Care Health Dev..

[28] Vander Zwart K.E., Geytenbeek J.J., de Kleijn M., Oostrom K.J., Gorter J.W., Hidecker M.J.C., Vermeulen R.J. (2016). Reliability of the Dutch-language Version of the Communication Function Classification System and Its Association with Language Comprehension and Method of Communication. Dev. Med. Child Neurol..

[29] Virella D., Pennington L., Andersen G.L., Andrada M.d.G., Greitane A., Himmelmann K., Prasauskiene A., Rackauskaite G., De La Cruz J., Colver A. (2016). Classification Systems of Communication for Use in Epidemiological Surveillance of Children with Cerebral Palsy. Dev. Med. Child Neurol..

[30] Tofani M., Mustari M., Tiozzo E., Dall’Oglio I., Morelli D., Gawronski O., Salata M., Cantonetti L., Castelli E., Di Lallo D. (2023). The Development of the International Classification of Functioning, Disability and Health for Child and Youth (ICF-CY) Core Sets: A Systematic Review. Disabil. Rehabil..

[31] Jeevanantham D., Dyszuk E., Bartlett D. (2015). The Manual Ability Classification System: A Scoping Review. Pediatr. Phys. Ther..

[32] Eliasson A.C., Krumlinde-Sundholm L., Rösblad B., Beckung E., Arner M., Öhrvall A.M., Rosenbaum P. (2006). The Manual Ability Classification System (MACS) for Children with Cerebral Palsy: Scale Development and Evidence of Validity and Reliability. Dev. Med. Child Neurol..

[33] Sellers D. (2018). Eating and Drinking Ability Classification System. Dysphagia.

[34] Tschirren L., Bauer S., Hanser C., Marsico P., Sellers D., van Hedel H.J.A. (2018). The Eating and Drinking Ability Classification System: Concurrent Validity and Reliability in Children with Cerebral Palsy. Dev. Med. Child Neurol..

[35] Sellers D., Pennington L., Bryant E., Benfer K., Weir K., Aboagye S., Morris C. (2022). Mini-EDACS: Development of the Eating and Drinking Ability Classification System for Young Children with Cerebral Palsy. Dev. Med. Child Neurol..

[36] Baranello G., Signorini S., Tinelli F., Guzzetta A., Pagliano E., Rossi A., Foscan M., Tramacere I., Romeo D.M.M., Ricci D. (2020). Visual Function Classification System for Children with Cerebral Palsy: Development and Validation. Dev. Med. Child Neurol..

